# A Case of an 80-Year-Old Man with Empyema and Psoas Abscess

**DOI:** 10.1155/2020/8895785

**Published:** 2020-10-24

**Authors:** Mikio Sakurai, Hiroki Nagasawa, Ikuto Takeuchi, Youichi Yanagawa

**Affiliations:** Department of Acute Critical Care Medicine, Shizuoka Hospital, Juntendo University, Japan

## Abstract

An 80-year-old man with flu symptoms collapsed at his house and had a backache worsened over time. His family called for an ambulance. On arrival, chest X-ray showed reduced permeability of the right lung field, and truncal computed tomography (CT) suggested right multilobular empyema and right iliopsoas abscess. A blood test showed an acute inflammatory response. The patient underwent right small thoracotomy for empyema and ultrasonic-guided drainage for the right iliopsoas abscess and started the administration of antibiotics. We started the administration of doripenem by intravenous drip and then deescalated to ampicillin based on the culture results. *Streptococcus intermedius* was cultured from all sites. Following these treatments for three months, his general condition improved. We herein report a unique case of complicated empyema and iliopsoas abscess in which a favorable outcome was obtained by an appropriate diagnosis and treatment. Reports of multiple abscesses have been increasing recently because of the growing geriatric population and aging-related complications. It is important to search the whole body to detect multiple abscesses in cases where an abscess is detected at a single site.

## 1. Introduction

Empyema is important historically that remains a modern menace. Causative bacteria include *Klebsiella pneumoniae*, *Streptococcus constellatus*, and *S. intermedius*, in decreasing order of frequency [1]. The prompt diagnosis and treatment with appropriate systemic antibiotics and chest tube drainage are the key management approaches [2].

Iliopsoas abscess is a rare condition with varied symptomology and etiology [3], leading to delays in the diagnosis and management, which can result in a fatal outcome [3].

We herein report a unique case of complicated empyema and iliopsoas abscess in which a favorable outcome was obtained by drainage and intravenous antibiotics.

## 2. Case History

An 80-year-old man with flu symptoms collapsed at his house because of lightheadedness. He received a diagnosis of lumbar compression fracture at a local clinic. He obtained nerve block injection to control his backache at another medical facility. However, he lost his appetite and could not stand, and his backache condition deteriorated, so his family called for an ambulance.

**Figure 1:**
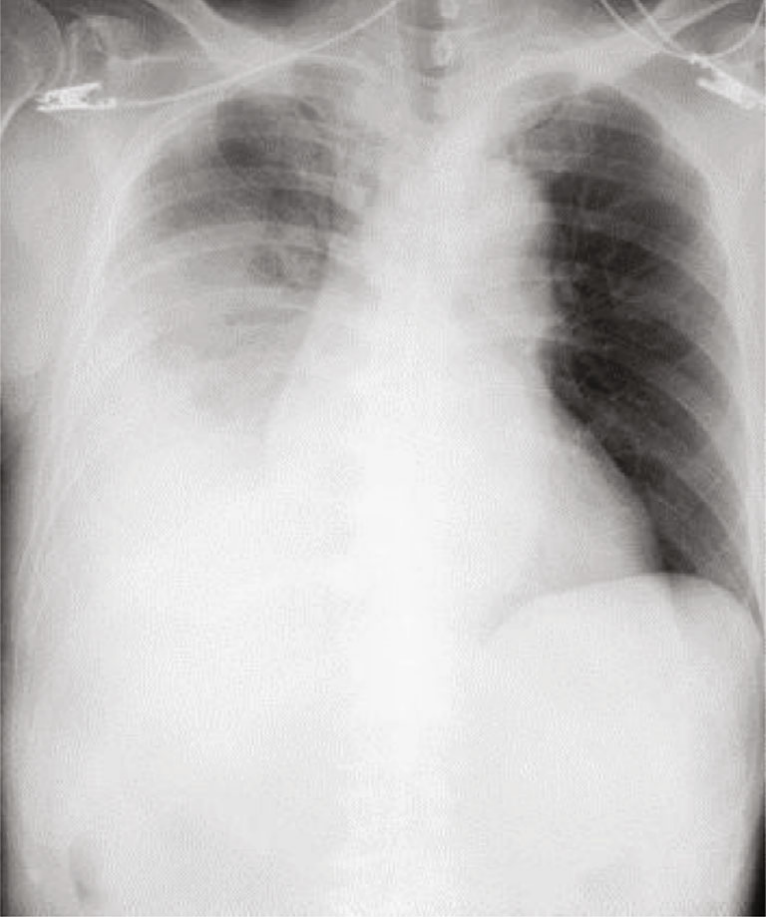
Chest X-ray findings on arrival. Chest X-ray showed reduced permeability of the right lung field.

**Figure 2:**
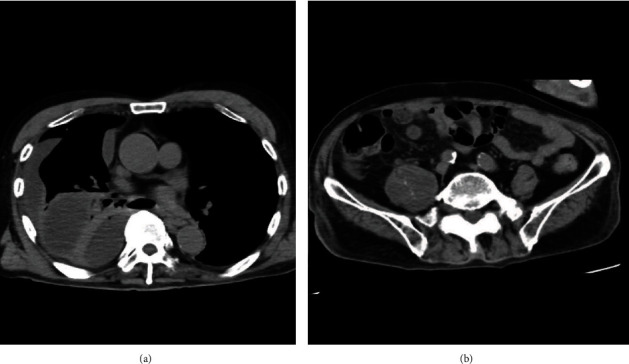
Truncal computed tomography (CT) findings on arrival. Truncal CT (axial section) suggested right multilobular empyema (a) and right iliopsoas abscess (b).

He had a history of gastrectomy at 31 years old, cerebral infarction at 71 years old, lumbar spinal canal stenosis at 73 years old, and acute coronary syndrome requiring percutaneous coronary intervention at 79 years old. He had no specific family history. On arrival, his blood pressure was 142/110 mmHg, his heart rate was 92 beats per minute, his body temperature was 37.2°C, and his percutaneous oxygen saturation was 97% under room air. He complained of backache on motion. He had no sensory or motor disturbance. The main results of a blood test are shown in Table 1. Chest X-ray showed reduced permeability of the right lung field (Figure 1), and truncal computed tomography (CT) suggested right multilobular empyema and right iliopsoas abscess (Figure 2). Exploratory puncture under ultrasonic guidance resulted in confirmation of the abscess, and we performed right small thoracotomy for empyema; about 300 ml of bloody pus was removed. We then inserted a continuous double lumen catheter into the right chest. Next, we performed drainage under ultrasonic guidance for the right iliopsoas abscess, and about 100 ml of bloody pus was also removed. We inserted a catheter for drainage of the remaining iliopsoas abscess. We diagnosed him with empyema with an iliopsoas abscess and started intravenous administration of doripenem hydrate after obtaining culture samples.

After these treatments, his general condition improved, and he was able to feed himself. On day 7, *S. intermedius* was cultured from all sites, and doripenem hydrate was deescalated to ampicillin based on the results of a drug sensitivity test and the possibility of switching to medicines for internal use. On day 9, all drainage tubes were removed because of an improvement in his inflammatory response. On day 16, lumbar fusion was performed for lumbar compression fracture. Following the operation, he continued rehabilitation and intravenous administration of ampicillin for three months. With these treatments, the abscess shrank. Finally, he was transferred to another hospital for rehabilitation.

## 3. Discussion

This is the unique case of a patient complicated with empyema and iliopsoas abscess who obtained a favorable outcome thanks to the appropriate diagnosis and treatment. There have been only two cases complicated with both empyema and iliopsoas abscess (Table 2) [4, 5]. Patients with abscess tend to have diabetes, chronic kidney disease, or immunodeficiency [4], but the present patient had none of these diseases.

Iliopsoas abscess is divided into primary and secondary types. Primary psoas abscess is presumed to arise via hematogenous or lymphatic spread, and *Staphylococcus aureus* is the causative bacteria in over 80% of cases [6]. Secondary psoas abscess is the consequence of the direct extension of infection around organs, most commonly Crohn's disease [7]. In the present patient, empyema and iliopsoas abscess were detected at the same time based on the causative bacteria, but we failed to identify any infectious sources near the iliopsoas muscle. Accordingly, the mechanism presumed to underlie the presence of abscesses at two different sites is as follows: aspiration pneumonia was the first infection induced by the aspiration of oral secretions. Next, the aspiration pneumonia resulted in the formation of empyema. Finally, bacteria in the empyema spread to the iliopsoas muscle hematogenously. This hypothesis is based on the fact that *S. intermedius* is an oral *Streptococcus* bacteria as well as a risk factor for aspiration pneumonia [8].

Reports of multiple abscesses have been increasing recently because of the growing geriatric population and aging-related complications. Accordingly, it is important to search the whole body via CT to detect multiple abscesses in cases where an abscess is detected at a single site [9].

## 4. Conclusion

This is the unique case of a patient complicated with empyema and iliopsoas abscess who obtained a favorable outcome by the appropriate diagnosis and treatment. Reports of multiple abscesses have been increasing recently because of the growing geriatric population and aging-related complications. It is important to search the whole body to detect multiple abscesses in cases where an abscess is detected at a single site.

## Figures and Tables

**Table 1:** Blood test findings on arrival.

White blood cell	13.5 × 10^9^/l
Hemoglobin	14.9 g/dl
Platelets	228 × 10^9^/l
Total protein	5.9 g/dl
Albumin	1.7 g/dl
Alkaline phosphatase	377 IU/l
Aspartate aminotransferase	85 IU/l
Alanine aminotransferase	78 IU/l
Lactate dehydrogenase	207 IU/l
*γ*-Glutamyl transpeptidase	22 IU/l
Cholinesterase	67 IU/l
Total bilirubin	1.1 mg/dl
Total cholesterol	99 mg/dl
Triglyceride	66 mg/dl
Blood urea nitrogen	14.8 mg/dl
Uric acid	2.4 mg/dl
Glucose	116 mg/dl
Creatinine	0.59 mg/dl
Amylase	34 IU/l
Creatine phosphokinase	14 IU/l
Sodium	128 mEq/l
Potassium	4.5 mEq/l
Chloride	90 mEq/l
Brain natriuretic peptide	40.4 pg/ml
Ammonia	13 pg/ml
Hemoglobin	A_1_C 5.8%
C-reactive protein	21.46 mg/dl
Prothrombin time	14.4 (13.3) sec
Activated partial thromboplastin time	28.5 (26.2) sec
Fibrinogen	727 mg/dl
Fibrinogen degradation products	8.5 *μ*g/ml

**Table 2:** The comparison of three cases.

Reporter	Age (years)	Sex	Primary disease	Causative bacteria	Treatment	Outcome
Present case	80	Male	None	*Streptococcus intermedius*	Antibiotics Drainage	Transfer
Liu et al. [4]	48	Male	Diabetes	*Streptococcus milleri*	Operation Antibiotics Drainage	Discharge
Ito and Miura [5]	78	Male	Diabetes	Negative	Antibiotics Drainage	Discharge

## References

[1] Chen K. Y., Hsueh P. R., Liaw Y. S., Yang P. C., Luh K. T. (2000). A 10-year experience with bacteriology of acute thoracic empyema. *Chest*.

[2] Brims F. J., Lansley S. M., Waterer G. W., Lee Y. C. G. (2010). Empyema thoracis: new insights into an old disease. *European Respiratory Review*.

[3] Shields D., Robinson P., Crowley T. P. (2012). Iliopsoas abscess--a review and update on the literature. *International Journal of Surgery*.

[4] Liu L., Goh Z. W., Rhodes B. (2013). Empyema and psoas abscess in a previously undiagnosed diabetic patient. *Journal of the New Zealand Medical Association*.

[5] Ito I., Miura A. (2011). A case of type 2 diabetes mellitus complicated with left iliopsoas abscess caused by a left femoral vein catheter during treatment for right pyothorax and right subphrenic abscess. *Nihon Ronen Igakkai zasshi. Japanese journal of geriatrics*.

[6] Ricci M. A., Rose F. B., Meyer K. K. (1986). Pyogenic psoas abscess: worldwide variations in etiology. *World Journal of Surgery*.

[7] Agrawal S. N., Dwivedi A. J., Khan M. (2002). Primary psoas abscess. *Digestive Diseases and Sciences*.

[8] Noguchi S., Yatera K., Kawanami T. (2015). The clinical features of respiratory infections caused by the *Streptococcus anginosus* group. *BMC Pulmonary Medicine*.

[9] Yanagawa Y., Aihara K., Watanabe S. (2013). Whole body CT for a patient with sepsis. *World Academy of Science, Engineering and Technology*.

